# Genome-scale DNA variant analysis and functional validation of a SNP underlying yellow fruit color in wild strawberry

**DOI:** 10.1038/srep29017

**Published:** 2016-07-05

**Authors:** Charles Hawkins, Julie Caruana, Erin Schiksnis, Zhongchi Liu

**Affiliations:** 1 Dept. of Cell Biology and Molecular Genetics, University of Maryland College Park, MD 20742, USA.

## Abstract

*Fragaria vesca* is a species of diploid strawberry being developed as a model for the octoploid garden strawberry. This work sequenced and compared the genomes of three *F. vesca* accessions: ‘Hawaii 4′, ‘Rügen’, and ‘Yellow Wonder’. Genome-scale analyses of shared and distinct SNPs among these three accessions have revealed that ‘Rügen’ and ‘Yellow Wonder’ are more similar to each other than they are to ‘Hawaii 4’. Though all three accessions are inbred seven generations, each accession still possesses extensive heterozygosity, highlighting the inherent differences between individual plants even of the same accession. The identification of the impact of each SNP as well as the large number of Indel markers provides a foundation for locating candidate mutations underlying phenotypic variations among these *F. vesca* accessions and for mapping new mutations generated through forward genetics screens. Through systematic analysis of SNP variants affecting genes in anthocyanin biosynthesis and regulation, a candidate SNP in *FveMYB10* was identified and then functionally confirmed to be responsible for the yellow color fruits made by many *F. vesca* accessions. As a whole, this study provides further resources for *F. vesca* and establishes a foundation for linking traits of economic importance to specific genes and variants.

*Fragaria vesca (F. vesca)* is a diploid species of wild strawberry that has been cultivated in European gardens for centuries. *F. vesca* is currently being developed as a model species for the garden strawberry^1^, *Fragaria x anannasa*, as well as the diverse *Rosaceae* family that includes apple, peach, almond and rose. The garden strawberry has a complex octoploid genome and *Rosaceae* fruit trees often need 3 to 7 years of juvenile growth before flowering, making them difficult systems for genetic studies. In contrast, *F. vesca* offers a number of advantages as a research model. First, the genome of a 4^th^-generation inbred line of *F. vesca*, Hawaii 4 × 4, has been sequenced and served as the reference genome. It consists of 240 MB spread across seven linkage groups (2n = 14)^1^. Second, *F. vesca* has a short life cycle of 4–6 months, is small in stature, is self-fertile, and is amenable to transformation. Finally, detailed morphological characterization and extensive flower and fruit developmental transcriptomes have been published for *F. vesca*, providing extensive resources to serve as a starting point for further studies^2^,^3^,^4^,^5^,^6^.

Three *Fragaria vesca* accessions (or varieties) have been developed for this purpose. They are ‘Hawaii 4′ (H4), ‘Rügen’ (Rü) (also referred to as ‘Ruegen’), and ‘Yellow Wonder’ (YW)^1^,^7^,^8^,^9^,^10^. The three accessions each have a 7^th^–generation inbred line, named H4 F7-3, Rügen F7-4, and YW5AF7 respectively. For simplicity, H4, Rü and YW will be used here to refer to the respective 7^th^-generation inbred lines. Although the three accessions are similar in their day-neutral flowering behavior (due to the *semperflorens* mutation) and in their physical stature and morphology, they also exhibit distinct characteristics. For example, H4 produces runners, a form of asexual reproduction, but YW and Rü do not (Fig. 1). In addition, Rü produces red fruit, while YW and H4 produce pale yellow fruit (Fig. 1). Classical genetic experiments have shown that both the runnerless trait and the yellow fruit color are each caused by recessive mutations at single loci^11^. Previous linkage analysis has shown that the *runnerless* locus maps to the end of LG2^12^. The same study, using a red fruited accession (Baron Solemacher) crossed with a yellow-fruited variety (Bush White), revealed that the locus responsible for the color difference (named locus *c*) is located near the end of Linkage Group 1 (LG1)^12^. Using intron length polymorphisms as markers and an F2 mapping population of YW (*F. vesca*) crossed with *F. nubicola* (FRA520), Deng and Davis observed co-segregation between an anthocyanin biosynthesis gene *Flavanone 3-Hydroxylase* (*F3H*) and the locus *c* and suggested *F3H* as a candidate gene for locus *c*^13^. However, up to now, the identity of locus *c* remains elusive.

The pigment that causes the characteristic red fruit color in strawberry is callistephin, a pelargonidin-based anthocyanin^14^,^15^,^16^. In addition to the enzymes that catalyze the synthesis of anthocyanins, several transcription factors have been shown to play critical roles in regulating anthocyanin production by controlling the expression of the biosynthesis genes. Chiefly, the MYB, bHLH, and WD-repeat proteins (which form the MBW complex) regulate the expression of the anthocyanin pathway genes in plants^17^,^18^. The most well-studied are the R2R3 MYB proteins; alteration in the expression or function of a single such MYB gene can drastically alter the accumulation of anthocyanins in orange^19^, petunia^20^, tomato^21^,^22^, and peach^23^. The garden strawberry homolog of *MYB10*, *FaMYB10*, was previously identified and shown to play critical roles in regulating the red pigment in the receptacle fruit. *FaMYB10* is specifically expressed during the ripening stage of the receptacle fruit, and its expression was shown to be repressed by auxin and stimulated by ABA^24^. Further, RNAi down-regulation of *FaMYB10* resulted in significant reduction of anthocyanin in the receptacle fruit mediated by the reduced expression of biosynthesis genes at both early and late stages in the anthocyanin biosynthesis pathway^24^. In contrast, over-expression of *FaMYB10* in the garden strawberry resulted in plants with elevated anthocyanin levels in roots, foliage, and fruit^25^. In *F. vesca*, an RNAi construct against *FveMYB10* was also shown to convert red fruit into pale yellow fruit while over-expression resulted in dark red fruits^26^, suggesting a similar role of *FveMYB10* in regulating red pigment synthesis in wild strawberry fruits.

In *F. vesca*, both red- and yellow- fruited accessions exist. Prior studies have shown that a high level of anthocyanins was present in the red fruit of ‘Rügen’ and ‘Reine des Vallees’ but not in the yellow fruit of ‘Yellow Wonder’^15^,^27^. Further, transcriptome profiling in red Rü and yellow YW receptacle fruit showed that transcript levels of several anthocyanin biosynthesis enzyme genes (C4H, CHS, CHI, F3H, DFR, and ANS) and several MYB genes (MYB1, MYB86 and MYB39) were reduced in the YW fruit^9^,^27^. However, these differentially expressed genes may reflect downstream effects of the causal mutation, and none of the studies conducted functional tests of DNA variants that may exist between yellow and red accessions.

In this study, we carried out genome-wide identification of variants among three inbred accessions of *F. vesca*, H4, YW, and Rü. Both SNP variants and structural variants that distinguish these three accessions were identified, which enables the development of molecular markers and will aid in gene mapping and gene isolation. Interestingly, although these three accessions all have previously been inbred for seven generations, each accession still possesses a large number of heterozygous loci. To identify the causal mutation that underlies the naturally occurring yellow fruit color in the *F. vesca* accessions H4 and YW, variants affecting exons of genes involved in anthocyanin biosynthesis and regulation were systematically examined and analyzed. Three SNPs affecting three different *FveMYB* transcription factors in the yellow fruit accessions were identified, and subsequent functional assays indicated that a single SNP causing an amino acid change (W12S) in the *FveMYB10* gene was responsible for the yellow color of these wild strawberry fruits.

## Results

### Genome-wide variant analyses reveal H4 as genetically more distinct from YW and Rü

To identify genome-wide variants among the three 7^th^ generation inbred accessions of *F. vesca*, H4, YW, and Rü, the genomic DNA of each was sequenced using the Illumina HiSeq2000 platform. Single-end, 51-bp reads were obtained for H4, YW, and Rü with 106, 84, and 80 million high quality reads, respectively (Supplementary Table S1). Subsequent sequence analyses (outlined in Supplementary Fig. S1) yielded a genome-wide total variant list with information on each SNP variant and its impact on previously-annotated genes (Supplementary Dataset S1). Supplementary Dataset S1 serves as the starting point for further filtering to identify accession-unique variants (Supplementary Dataset S2), heterozygous variants (Supplementary Dataset S3), and “high impact” variants in each of the above two categories (Supplementary Datasets S1, S2, S3).

The “accession-unique variants” (Supplementary Dataset S2) allow us to investigate the relationships among the three accessions. Variants are considered unique to an accession if they are present in one accession but absent from the other two. An accession-unique variant may either exist as homozygous or heterozygous within the accession. We found that H4 has 99,722 unique loci, Rü has 38,404 unique loci, and YW has 42,483 unique loci (Supplementary Fig. S2). Additionally, YW and Rü have more SNPs in common (Supplementary Fig. S2), indicating that YW and Rü are more similar to each other than they are to H4.

**Figure 2:**
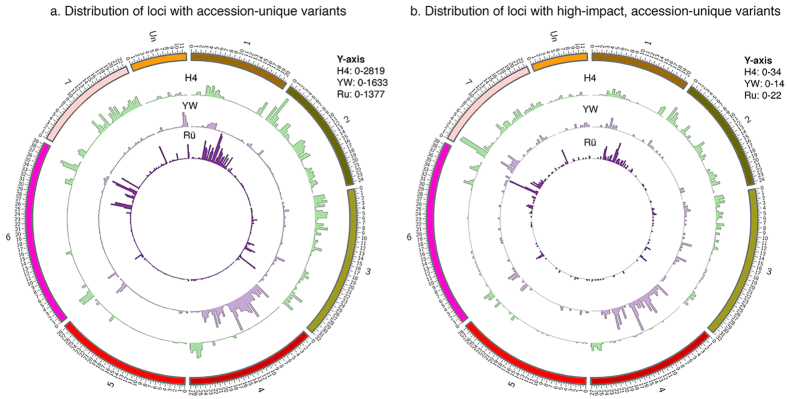
Genome distribution of accession-unique variants and unique high impact variants. (**a**) Circos histograms showing distribution of variants unique to each accession. (**b**) Circos histograms showing high impact variants unique to each accession. Bin size is 500 kb, Y-axis is the number of unique or high-impact/unique variants in that bin. The outermost track represents the seven *F. vesca* Linkage Groups plus the unanchored scaffolds (Un), drawn to scale in Mbp.

We plotted the density of accession-unique loci along each of the seven Linkage Groups (LGs; i.e., chromosomes) (Fig. 2A). There is considerable variation in the distribution of these loci. LGs 2, 3, and 7 are particularly rich in H4-unique variants, suggesting independent origins of these three LGs in H4. This is consistent with the runner locus being previously mapped to LG2^12^, as H4 is the only accession here that exhibits the runnering trait. Next, LG1 is the only LG rich in variants unique to Rü. This is consistent with LG1 containing the color locus *c*^12^ as Rü is the only accession here that carries the red allele. Third, LG4 is rich in SNPs unique to YW. All five of the other chromosomes (LG2, 3, 5, 6, 7) are largely shared between Rü and YW (Fig. 2A). Finally, LG5, LG6, and a roughly 5 Mb stretch of LG7 contain very few loci unique to any accession; the total combined variants (unique and shared) are relatively low in these regions as well (Supplementary Fig. S3).

### Analysis of the impact of each variant on protein coding genes

In order to determine the impact of each SNP on protein coding genes, we utilized the snpEff v4.0 program. Of the 366,057 total variants annotated by snpEff v4.0 (Supplementary Dataset S1), 310,266 were found to be within 5 kb of at least one gene, 106,862 are within introns, 14,220 are silent coding sequence variants, and 26,437 have “high to moderate impacts” on the coding sequence (Supplementary Dataset S1). Moderate-impact variants are single-residue missense variants that do not affect the start or stop of translation. In contrast, high impact variants are those whose changes to the coding sequence may result in a knockout of the affected gene. They include frameshifts, loss of “start”, loss or gain of “stop”, and loss or gain of splice sites. 3,030 were categorized as high-impact variants (Supplementary Dataset S1). High-impact variants unique to each accession were also extracted and plotted across all linkage groups (Fig. 2B; Supplementary Dataset S2). Although these high-impact variants are significantly lower in number, their distribution pattern largely mirrors the unique variant distribution (Fig. 2A) except that the beginning of LG7 is abundant with high impact variants unique to H4.

Gene Ontology (GO)^28^ enrichment analysis was performed on the total combined high impact variants using Blast2GO. The enriched GO categories were primarily related to DNA binding and DNA replication (Supplementary Fig. S4). Other enriched categories appear to be related to RNA-dependent DNA replication.

### All three varieties exhibit a high level of heterozygosity

**Figure 3:**
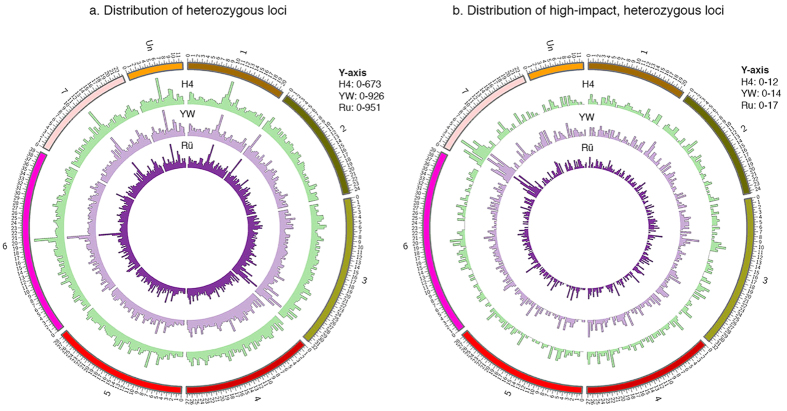
Genome distribution of heterozygous variants and heterozygous high impact variants. (**a**) Circos histogram showing distribution of heterozygous loci in each accession. (**b**) Circos histogram showing distribution of heterozygous and high impact loci in each accession. Bin size is 500 kb, Y-axis is the number of heterozygous or high-impact/heterozygous variants in that bin. The outermost circle represents the seven *F. vesca* Linkage Groups plus the unanchored scaffolds (Un), drawn to scale in Mbp.

A highly homozygous genome is desirable for molecular genetic studies. While the reference genome derived from H4 × 4 consists of a single sequence file, we found that multiple alternative bases existed in the respective genomes of H4, YW, and Rü inbred lines (Supplementary Dataset S1). A significant number of loci in the genomes of all three accessions were heterozygous, with H4 showing 90,453 such loci, Rü showing 119,693 heterozygous loci, and YW showing 121,789 heterozygous loci (Supplementary Fig. S2; Supplementary Dataset S3). The density of these heterozygous SNPs across the genome was plotted (Fig. 3A). Across most of the genome, heterozygosity is similar among the three accessions. However, H4 shows lower levels of heterozygosity than the other two in some regions, most notably at the end of LG3 and at the beginning of LG2 (Fig. 3A). On the other hand, YW shows slightly higher levels of heterozygosity in LG4 than the other two accessions.

One hypothesis for the persistence of heterozygosity in these 7^th^-generation inbred lines is that heterozygosity could balance high impact SNPs and prevent homozygous lethality. We extracted heterozygous loci from the high impact variant list (Supplementary Dataset S1) to yield heterozygous high impact variants (Supplementary Dataset S3). We then plotted them across the seven LGs (Fig. 3B). Compared with total heterozygous loci (Fig. 3A), the high impact heterozygous variants are significantly lower in number but nevertheless are distributed throughout all seven LGs (Fig. 3B); only LG7 shows a peak region near the beginning of the chromosome. GO term analysis of high impact heterozygous variants could not identify statistically significant categories due to the small number of genes. Subsequently, we examined the percentage of high impact SNPs among all SNPs vs. the percentage of high impact SNPs among heterozygous loci. 0.87% of all loci were found to be high-impact, while 0.73%, 0.79%, and 0.76% of heterozygous loci in H4, Rü, and YW respectively are high-impact. The data indicate that there is no enrichment of high impact SNPs among heterozygous loci, indicating that our prior hypothesis is incorrect.

### Identification of Indels and structural variants

In addition to SNP variants, we conducted genome-wide detection of large insertions and deletions (Indels) (Supplementary Dataset S4) and structural variants (Supplementary Dataset S5) in the three accessions using CLC Genomics Workbench. Because of the short read length of 51 bp and because the reads are unpaired, many large structural variants may be missed. Nevertheless, we developed three accession-specific markers based on the Indel information (Supplementary Dataset S4). Each Indel marker can distinguish one of the three accessions based on a simple PCR reaction (Supplementary Fig. S5). Together, this set of Indel markers can easily distinguish each of the three accessions and can be used to confirm and distinguish hybrids among these three accessions. The variant lists (Supplementary Dataset S2 for SNPs, Supplementary Dataset S4 for Indels, Supplementary Dataset S5 for structural variants) provide useful information for the development of region-specific markers for gene mapping and cloning.

### Identification of candidate SNPs responsible for the yellow fruit color

**Figure 4:**
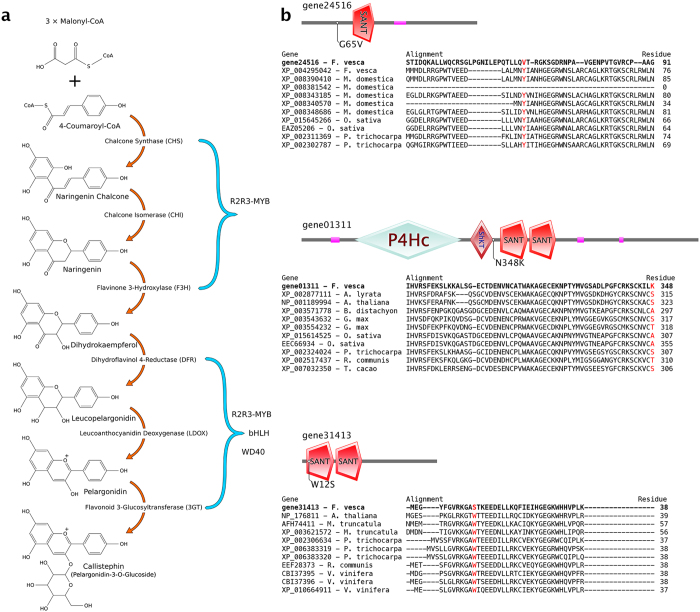
Canonical anthocyanin biosynthesis pathway and sequence alignment of candidate MYB genes. (**a**) Canonical anthocyanin biosynthesis pathway showing enzymes and intermediate pigments in each step. Steps regulated by R2-R3 MYB transcription factors are also indicated. This pathway illustration is based on a published paper^15^,^29^. (**b**) MYB protein domain structure and the location of the variant in each of the candidate *FveMYB* genes. Sequence alignment of the protein region affected by the concerned variant (red) is shown for each *MYB* gene.

**Figure 5:**
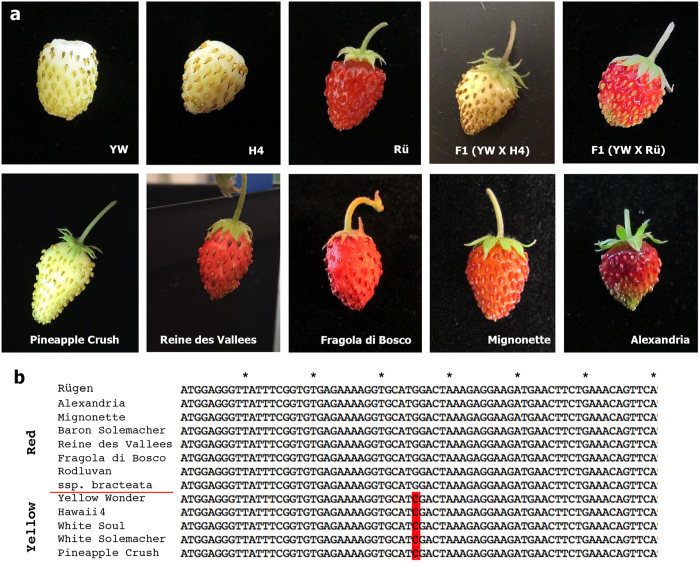
Genetics of fruit color trait and survey of the *FveMYB10* W12S variant in red and yellow *F. vesca* accessions. (**a**) Fruit phenotype from different *F. vesca* accessions including F1 progeny of crosses between red and yellow accessions. (**b**) Sequence of a segment of *FveMYB10* DNA in red and yellow fruit accessions. The specific SNP (G to C) that converts the W to S at position 12 is highlighted. All red accessions have G, while all yellow accessions have C.

The red fruit pigment in strawberry is Callistephin (Pelargonidin-3-O-Glucoside), the biosynthetic pathway of which is well established (Fig. 4A)^14^,^15^,^29^. We investigated the molecular basis underlying the red vs yellow fleshy fruit in these three accessions. Red-fruited Rü was crossed to yellow-fruited YW, and the F1 progeny produced red-fruit (Fig. 5A). This is consistent with previous genetic evidence^11^ and indicates that the red color is dominant and the yellow color likely results from a loss-of-function mutation. A cross between the two yellow accessions (H4 and YW) resulted in yellow fruit (Fig. 5A), indicating that H4 and YW are defective in the same gene as they failed to complement each other.

To identify a potential SNP responsible for the yellow fruit color, we first assembled a short list of likely candidate genes based on prior knowledge of fruit pigment biosynthesis and regulation including genes coding for enzymes of pelargonidin biosynthesis as well as the R2R3 MYB-class transcription factors (Fig. 4A; Supplementary Dataset S6). Then, we searched for variants that are of high or moderate impact as well as unique and homozygous in Rü. The resulting variant list was intersected with the pigment biosynthesis and regulation gene list (Supplementary Dataset S6). Manual filtering by checking mapped reads and determining homozygosity resulted in eight SNPs (Supplementary Table S2); two reside in 3GT (Flavonoid 3-Glucosyltransferase) genes, two in MYB-like or MYB-related genes, and four reside in the *FveMYB* genes. Among these eight genes, only three *FveMYB* genes possess variants that cause amino acid substitutions from one property type to another (Supplementary Table S2). Specifically, gene01311 has an Asn (N) to Lys (K) substitution (polar to basic) at position 348, gene24516 has a Gly (G) to Val (V) substitution (polar to nonpolar) at position 65, and finally gene31413 exhibits a Trp (W) to Ser (S) substitution (nonpolar to polar) at position 12. To narrow down which SNP is more likely the candidate SNP, we sought to determine if any of the SNPs affects a conserved residue (Fig. 4B). Neither the SNP in gene01311 nor the SNP in gene24516 affects a conserved residue. In contrast, the SNP in gene31413 affects a highly conserved residue W within the R2 DNA-binding domain (Fig. 4B). In addition, gene31413 was previously named as *FveMYB10* and shown by RNAi to cause yellow fleshy fruit in *F. vesca*^26^. Therefore, the specific W12S SNP in gene31413 (*FveMYB10*) emerged as the primary candidate for causing the yellow fruit color in the woodland strawberry.

### Experimental test of the effect of W12S on fruit color

We first tested whether the W12S variant is tightly associated with the yellow fruit color in three other yellow-fruited *F. vesca* accessions: White Soul, Pineapple Crush, and White Solemacher. Pair-wise genetic crosses were made between H4 and each of these three yellow fruited accessions. The F1 progeny all produced yellow fruits, indicating that these yellow accessions are all defective in the same gene as H4. PCR amplification and sequencing of the *FveMYB10* gene from these additional yellow-fruited accessions revealed that all of them had the C nucleotide and thus the W12S substitution in *FveMYB10* (Fig. 5B). Seven additional accessions or subspecies that produced red fruits were similarly analyzed; they were Alexandria, Mignonette, Baron Solemacher, Reine des Vallees, Fragola di Bosco, Rodluvan, and *ssp*. bracteata. All of these red-fruited accessions/subspecies were shown to possess the wild type G nucleotide in *FveMYB10* (Fig. 5B). Hence, among the yellow and red-fruited varieties tested, the C (W12S) in *FveMYB10* is 100% associated with the yellow fruit color, while the G is 100% associated with the red fruit color.

**Figure 6:**
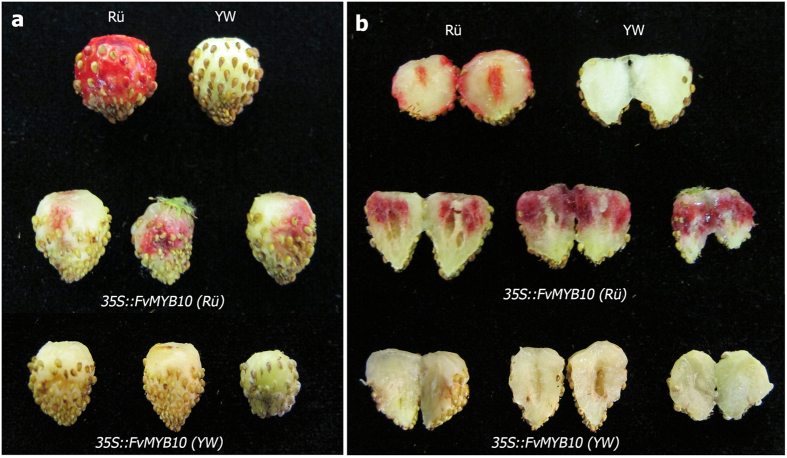
Functional test of the *FveMYB10* W12S variant in fruit color determination. (**a**) Images of control fruits (top row) and fruits of YW injected with *Agrobacterium* containing the *35S::FveMYB10 (Rü)* (middle row) or *35S::FveMYB10(YW)* construct (bottom row). Three representative fruits from each construct are shown. (**b**) Same fruits from (**a**) were cut in half to reveal their interior color.

Next we carried out a functional assay to test the ability of *FveMYB10* to restore red color in the YW yellow fruit. Full-length *FveMYB10* cDNAs were isolated from Rü (red) and YW (yellow), respectively. Sequence analysis confirmed that the only difference between these two *FveMYB10* cDNAs is the G in the Rü (red) cDNA and C in the YW (yellow) cDNA. Subsequently, the Rü and YW cDNAs were respectively cloned behind the 35S promoter in the pMDC32 vector. *Agrobacterium tumefaciens* containing each construct was injected into the developing fruit of YW. Out of nineteen yellow fruits injected with the *Agrobacterium* containing *35S::FveMYB10(Rü)*, sixteen developed red pigmentation at the injection sites (Fig. 6A,B) and three did not survive. Of the twelve YW fruits injected with *Agrobacterium* containing *35S::FveMYB10(YW)*, 10 survived the treatment but none developed red pigment (Fig. 6A,B). As the only difference between the Rü cDNA and YW cDNA is the single SNP, we concluded that the G to C change (W12S substitution) renders *FveMYB10* nonfunctional and determines the yellow fruit color in many of the wild *F. vesca* accessions.

## Discussion

While the exact history and origin of the three *F. vesca* accessions under study are unclear, all three are perpetual flowering (*F. vesca semperflorens*). The perpetual flowering *F. vesca* was described in 1766 and introduced into gardens all over Europe at that time^30^,^31^,^32^. The specific mutation responsible for the perpetual flowering was recently shown to result from a 2 bp deletion in the *FveTFL* gene^28^,^29^. To date, H4, YW, and Rü are frequently used in research due to the availability of the H4 genome sequence and the continuous flowering habit.

Genome-wide identification and analysis of variants among the three varieties provided detailed information on SNP variants (Supplementary Dataset S1), Indels (Supplementary Dataset S4), and structural variants (Supplementary Dataset S5). Combined, they will greatly aid in gene mapping and gene isolation. In addition, determination of total and accession-unique high impact variants (Supplementary Datasets S1 and S2) provides the starting point to examine genetic changes between varieties and the resulting phenotypic differences. The availability of the genome sequence reads of the three accessions submitted to NCBI-SRA (SRP068157) and the information about their heterozygosity will be of significant interest to researchers wishing to use *F. vesca* to map and isolate genes. Together, the various genomic analysis data reported here help further establish *F. vesca* as a model system.

One surprising finding from this study is the high level of heterozygosity for all three accessions despite each being inbred for seven generations. Specifically, Rü has 119,693 heterozygous loci, YW has 121,789 heterozygous loci, and H4 has 90,453 heterozygous loci (Supplementary Fig. S2). The observed heterozygosity may even be an underestimate due to the pooling of only two individual plants per accession for sequencing. Heterozygosity was reported for the soybean reference cultivar Williams 82 after six generations of testcross followed by one generation of self cross^33^. However, about 1800 heterozygous loci were found in Williams 82. Theoretically, every generation of inbreeding should reduce heterozygosity by half; 7^th^ generation inbred plants should have reduced heterozygosity by 128 times compared to the progenitor plant. With the current number of approximately 100,000 heterozygous loci spread across the 200 Mb genome (ie. one heterozygous locus every 2 kb), the original strain would be predicted to have one heterozygous variant every 16 bp (2 kb divided by 128), which seems too high. An alternative and more reasonable interpretation is that the inbreeding process has not been efficient and did not cut down heterozygosity by half each generation as predicted. Another possibility is that unintended cross-pollination among sibling plants may have occurred during the inbreeding regimes, slowing the progress toward homozygosity. Third, heterozygosity may offer significant hybrid vigor that could be selected for during the inbreeding process. Finally, though not supported by our data, heterozygosity may buffer deleterious effects caused by the high impact SNPs.

The high level of heterozygosity in *F. vesca* accessions raises the question of which nucleotide, for each heterozygous locus, is represented by the reference genome^1^. As a consequence, the inherent differences between individual plants should always be considered when utilizing the reference genome to design PCR primers, develop molecular markers, conduct sequence comparisons, and perform BSA (Bulk-Segregant-Analysis)-Seq. For example, a mapping population developed from a single F1 individual is advisable when conducting BSA-Seq, and analysis filters should be used to remove heterozygous SNPs before gene mapping during BSA-Seq. To further the usefulness of *F. vesca* as a model plant, it may be necessary to develop double haploid lines, perhaps by applying the CENH3-based haploid inducers^34^.

Although *MYB10* has previously been shown to regulate red pigment synthesis in both diploid and octoploid strawberry fruits^24^,^25^,^26^, it is not known if the yellow fruit made by *F. vesca* is caused by a defective *FveMYB10* gene. Mutations in genes that regulate *FveMYB10* or genes regulated by *FveMYB10* could also lead to yellow fruit in these *F. vesca* accessions. Therefore, a systematic search of potential candidate genes was necessary, which led to three *FveMYB* genes harboring nonsynonymous mutations (Supplementary Table S2). However, only the SNP in *FveMYB10* affected the conserved DNA-binding domain (Fig. 4B) and thus emerged as the primary candidate. Given that RNAi-mediated knockdown of *FveMYB10* was sufficient to cause yellow fruit^26^, a demonstration of a non-functional FveMYB10 (W12S) in YW should provide strong evidence for this variant as the causal mutation leading to the yellow fleshy fruit. Our transient functional assay clearly showed that this W12S in *FveMYB10* abolished *FveMYB10’s* ability to restore red pigment in the yellow fruit of YW. Interestingly, we observed that the interior receptacle tissue developed red color after being injected with Agrobacterium containing the *35S::FveMYB10(Rü)* construct (Fig. 6B) despite the fact that Rü normally does not develop red pigment there. It is likely that *FveMYB10* is normally expressed only in the outer surface of the receptacle, while ectopic *FveMYB10* expression driven by the 35S promoter in the injected portion of the receptacle was responsible for the interior red color.

Additional biochemical and genetic evidence supports a causal role for W12S FveMYB10 in yellow fruit color. In previous biochemical and structural studies of MYB proteins in animals, each of the three conserved tryptophan (W) residues within the R domain was shown to be critical in maintaining the hydrophobic core of the R-domain and hence its DNA-binding function^35^,^36^. Therefore, the W (hydrophobic) to S (polar) change in the R2 DNA-binding domain of FveMYB10 likely disrupts the DNA-binding function of R2. Second, previous genetic mapping showed that the fruit color locus c in Bush White (another yellow-fruited accession) resides on LG1^12^. Consistent with the genetic mapping of the locus *c* to LG1, genome sequencing and annotation also placed *FveMYB10/*gene31413 on LG1 (between 15,405,782 bp and 15,407,498 bp). The FveMYB10 (W12S) variant most likely arose from a spontaneous mutation in an ancestral *F. vesca* that gave rise to the yellow colored fruits of many *F. vesca* accessions. Given that red fruit is important for attracting birds and other animals for seed dispersal^37^, it is intriguing that the yellow-fruited *F. vesca* accessions co-exist with the red accessions in nature. Our work reported here provides an example of utilizing genomic comparisons to connect traits of economic importance to specific genes and variants.

## Methods

### Plant materials and DNA sequencing

A 7th generation inbred line of Hawaii 4 (H4 F7-3), National Germplasm Repository ID PI664444, was used in this study. Similarly, a previously described 7th generation inbred line of Rügen (Rü F7-4)^8^,^9^ was used. Yellow Wonder 5AF7 (PI641092) is also a 7th generation inbred line described previously^4^,^7^. Other strawberry accessions shown in Fig. 5 were purchased from The Strawberry Store (http://thestrawberrystore.com); these varieties were verified for their fruit color phenotype and were crossed with H4 or YW for complementation tests on the fruit color trait. For DNA sequencing, young leaves were harvested from two individual plants of each variety, and genomic DNA was isolated using the NucleoSpin Plant II kit (Macherey-Nagel, Duren, Germany). DNA samples were mailed to the Genomics Research Core Facility at the Weill Cornell Medical College for library preparation and sequencing on the Illumina HiSeq2000 instrument. The resulting single-end, 51-bp read statistics are summarized in Supplementary Table S1.

For sequence analysis of *FveMYB10* from yellow and red strawberry varieties shown in Fig. 5, the gene fragment was amplified using Phusion High-Fidelity DNA Polymerase (New England Biolabs, Ipswich, MA) with primers 5′ GCTCAAATATAGGTAACGTCAATACTC 3′ (forward) and 5′ CAGACGTAAACATACATATAAGCAGC 3′ (reverse). PCR fragments were purified using the NucleoSpin Gel and PCR Clean-up kit (Macherey-Nagel, Duren, Germany) and sequenced at Macrogen (Rockville, MD).

### Variant analysis pipeline

The variant analysis pipeline is summarized in Supplementary Fig. S1. Briefly, raw sequence reads were mapped to the reference genome of H4 × 4, genome-wide SNP variants were called through the GATK program^38^, with a filter passing variants with a Phred-scaled quality score over 30. The resulting SNP variant list was further annotated by the snpEff v4.0 program^39^ to generate an annotated total SNP variant list (Supplementary Dataset S1).

Specifically, the reference genome of *F. vesca* Hawaii 4 (version 1.1) and the genome annotation file (Fvesca_226_gene.gff3) were downloaded from Phytozome (phytozome.jgi.doe.gov)^40^. Raw sequence reads were mapped to the reference genome using BWA (Burrows-Wheeler Aligner)^41^ after indexing with Picard (http://broadinstitute.github.io/picard/) and SAMtools^42^. The GATK software package (www.broadinstitute.org/gatk/)^35^ was used for variant detection. The genome annotation file (Fvesca_226_gene.gff3) representing 32,831 genes needed modification to fix the exon offsets using the custom script “fix-gff.awk” (Supplementary Methods). The modified gene annotation list and the GATK-derived raw variant list were fed into snpEff v4.0 (snpeff.sourceforge.net)^39^, which predicted the impact of variants on protein-coding genes and yielded the annotated variant list, in which each variant was noted for its impact.

Two filtering steps were then applied to the annotated variant list to eliminate variants with low quality reads as well as variants with reads >50 as regions with abnormally high read abundance may reflect repetitive or questionable regions. To achieve this, BedTools^43^ was used to create a map of read depth across the genome, custom script “extractHCRanges.awk” (Supplementary Methods) was used to mark regions with reads >50, and custom script “cutReads” (Supplementary Methods) was used to remove all SNPs located in such regions (reads >50). This filtering step led to the final variant list (Supplementary Dataset S1). Subsequently, moderate and high impact variants were extracted from the annotated variant list using the “grep” unix command. After the same filtering steps (described above) that eliminate low quality reads and reads >50, the moderate and high impact variants are shown in separate sheets of Supplementary Dataset S1.

To identify variants unique to each accession, a custom Awk script “uniqueVariants.awk” (Supplementary Methods) was used to filter the annotated variant list, yielding variant unique to each of the three accessions (Supplementary Dataset S2). ClicO FS (Circular Layout Interactive Converter Free Services), a web-based interface incorporating the Circos circular genome visualization tool^44^,^45^, was used to plot accession-unique SNP variants shown in Fig. 2A.

To identify SNP variants heterozygous in each of the accessions, a custom script “heterozygousVariants.awk” (Supplementary Methods) was used that yielded Supplementary Dataset S3. Since two individual plants (four haploid genomes) of each cultivar were pooled and sequenced, a locus was judged heterozygous in an accession if GATK found more than one allele at that locus within the pool of four haploid genomes. The clicO FS web tool was used to plot heterozygous SNP variants shown in Fig. 3A.

Finally, the high impact variants identified above were filtered using custom script “uniqueVariants.awk” to generate high impact variants that is also unique to each accession (Supplementary Dataset S2 as separate sheets). Similarly, the high impact variants were filtered via script “heterozygousVariants.awk” to generate high impact and heterozygous variants (Supplementary Dataset S3 as separate sheets). To generate Supplementary Datasets S2 and S3, we removed SNPs located in regions with reads >50 or reads of low quality as we did with Supplementary Dataset S1. The clicO FS web tool was used to plot “high impact unique variants” and “high impact heterozygous variants” in Figs 2B and 3B, respectively.

Several sets of genes were tested for enriched GO categories using Fisher’s Exact Test (p < 0.05, FDR filtered) performed by the Blast2GO software v3.1.3^28^. Results were reduced to the most specific categories. The set of genes with high-impact variants was tested against the set of all annotated genes in the genome (Supplementary Fig. S3).

### Identification of potential variants responsible for the lack of anthocyanin in fruits of YW and H4

Members of anthocyanin biosynthetic genes were identified by their annotation in the Plaza 2.5 database (http://bioinformatics.psb.ugent.be/plaza/)^46^. The *MYB* family transcription factors were extracted from the published list of transcription factor genes in *F. vesca*^6^. The resulting combined list is Supplementary Dataset S6.

To identify the candidate mutation responsible for the yellow fruit color in YW and H4, SNPs that are unique in Rü were identified from Supplementary Dataset S2. Next, SNPs with nonsynonymous changes in protein-coding regions (marked as “MODERATE” or “HIGH” impact by snpEff) were identified from Supplementary Dataset S1. Subsequently, SNPs fulfilling both of the above requirements and at the same time residing in one of the anthocyanin gene list (Supplementary Dataset S6) were identified. There were only eight genes affected by such a SNP (Supplementary Table S2). For sequence alignment (Fig. 5B), each of the three *FveMYB* candidate genes served as the query in blastp within Plaza 2.5. Each hit was then identified with an NCBI accession using NCBI BLAST. The top 10 hits that could be identified were aligned with the query using ClustalX. Each gene structure was drawn based on diagrams from SMART (http://smart.embl-heidelberg.de/)^47^.

### Identification of indels and structural variants and PCR genotyping

To identify indel variants larger than 10 bp in length (the upper limit for GATK), we used the “Indels and Structural Variants” tool in the CLC Genomics Workbench. Illumina reads were first mapped to the *F. vesca* genome (version 2.0.a1)^48^ using the following parameters: mismatch cost −2, insertion cost −3, deletion cost −3, length fraction −0.5, similarity cost −0.9, no global alignment, and non-specific matches were mapped randomly. Next, the “InDels and Structural Variants” tool was used with default parameters to identify large variants shown in Supplementary Dataset S4 and S5.

Based on the Indel variant table (Supplementary Dataset S4), we identified potential indel markers that are unique in at least one accession. Primer sequences and amplicon lengths for each genotype are shown in Supplementary Table S3. PCR was carried out using AccuStart™ II PCR ToughMix (Quanta Biosciences; Gaithersburg, MD) at the following reaction conditions: 94 °C for 3 min initial denaturation, 35 cycles of [94 °C for 30 sec, 58 °C for 30 sec, 72 °C for 30 sec], and final extension of 72 °C for 5 min. PCR products were visualized on 1% agarose gel using DNA SafeStain (Lamda Biotech, St. Louis MO).

### Plasmid construction and functional assays in fruits

RNA was isolated from “white” stage (19 days post-anthesis)^6^ Rü and YW fruits using the Qiagen RNEasy Plant Mini kit (Qiagen). Approximately 200 ng of total RNA was used to generate cDNA using the iScript cDNA Synthesis kit (Bio-Rad). The *FveMYB10* coding sequence was PCR amplified from both Rü and YW cDNA using primers 5′ATGGAGGGTTATTTCGGTGTGAG 3′ (forward) and 5′ TACGTAGGAGATGTTGACTAGATCATTGC 3′ (reverse) and cloned using the pCR™8/GW/TOPO TA Cloning Kit (Invitrogen). After sequencing to confirm the correct CDS sequence for both Rü and YW clones, each was independently recombined into the pMDC32 binary vector^49^ using the Gateway LR Clonase II Enzyme mix (Invitrogen) to produce the overexpression vectors.

The transient expression assay in *F. vesca* fruits was carried out according to the procedure used for *F. x ananassa*^50^ with slight modifications. *Agrobacterium tumefaciens* strain GV3101 containing the 35S::*FveMYB10* construct was grown overnight to an O.D. of 0.8–1.0, then resuspended at a final O.D. of 0.8 in liquid MS medium containing 2% sucrose. A sterile 1 mL hypodermic syringe was used to evenly inject the suspension into the cortex of the fruits. Fruits ranging from “white” to “turning” (22 days post anthesis) stages were injected once and were left on the plants to continue growth. Patches of red color appeared 3–14 days after injection, with younger fruits taking longer to show red color. Mature, ripe fruits were not suitable for injection.

## Additional Information

**Accession codes:** Raw Illumina DNA-sequencing files of Rügen F7-4, YW5AF7, and H4 F7-3 have been submitted to NCBI SRA with the accession number SRP068157.

**How to cite this article**: Hawkins, C. *et al*. Genome-scale DNA variant analysis and functional validation of a SNP underlying yellow fruit color in wild strawberry. *Sci. Rep*. **6**, 29017; doi: 10.1038/srep29017 (2016).

## Supplementary Material

Supplementary Information

Supplementary Dataset S1

Supplementary Dataset S2

Supplementary Dataset S3

Supplementary Dataset S4

Supplementary Dataset S5

Supplementary Dataset S6

## Figures and Tables

**Figure 1:**
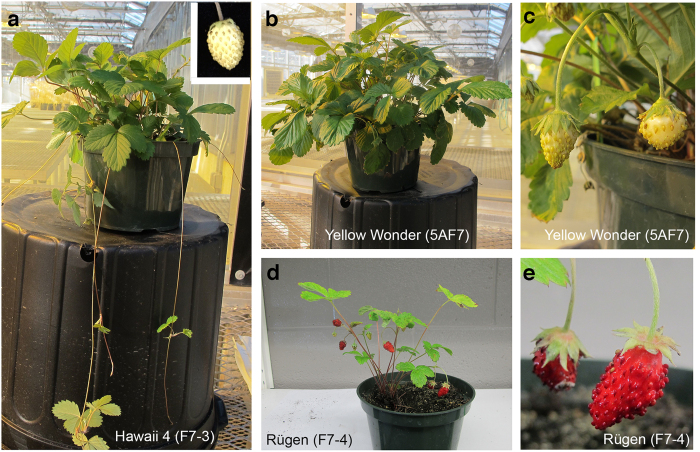
Three *F. vesca* accessions used in this study. (**a**) H4 plant showing runners and yellow fruit (inset). (**b**) YW plant showing a lack of runners. (**c**) A YW plant with yellow fruits. (**d**) Rü plant showing red fruits and a lack of runners. (**e**) Enlarged image of the red fruit from Rü.

## References

[b1] Shulaev V. . The genome of woodland strawberry (Fragaria vesca). Nat Genet 43, 109–16 (2011). 21186353 10.1038/ng.740 PMC3326587

[b2] Darwish O. . SGR: an online genomic resource for the woodland strawberry. BMC Plant Biol 13, 223 (2013). 24364888 10.1186/1471-2229-13-223 PMC3878773

[b3] Darwish O., Shahan R., Liu Z., Slovin J. P. & Alkharouf N. W. Re-annotation of the woodland strawberry (Fragaria vesca) genome. BMC Genomics 16, 29 (2015). 25623424 10.1186/s12864-015-1221-1 PMC4318131

[b4] Hollender C. A., Geretz A. C., Slovin J. P. & Liu Z. Flower and early fruit development in a diploid strawberry, Fragaria vesca. Planta 235, 1123–39 (2012). 22198460 10.1007/s00425-011-1562-1

[b5] Hollender C. A. . Floral transcriptomes in woodland strawberry uncover developing receptacle and anther gene networks. Plant Physiol 165, 1062–1075 (2014). 24828307 10.1104/pp.114.237529 PMC4081322

[b6] Kang C. . Genome-scale transcriptomic insights into early-stage fruit development in woodland strawberry Fragaria vesca. Plant Cell 25, 1960–78 (2013). 23898027 10.1105/tpc.113.111732 PMC3723606

[b7] Slovin J. P., Schmitt K. & Folta K. M. An inbred line of the diploid strawberry Fragaria vesca f. semperflorens for genomic and molecular genetic studies in the Rosaceae. Plant Methods 5, 15 (2009). 19878589 10.1186/1746-4811-5-15 PMC2780397

[b8] Sun J., Liu X., Yang T., Slovin J. & Chen P. Profiling polyphenols of two diploid strawberry (Fragaria vesca) inbred lines using UHPLC-HRMS(n.). Food Chem 146, 289–98 (2014). 24176345 10.1016/j.foodchem.2013.08.089 PMC3902803

[b9] Xu W. . Effect of calcium on strawberry fruit flavonoid pathway gene expression and anthocyanin accumulation. Plant Physiol Biochem 82, 289–98 (2014). 25036468 10.1016/j.plaphy.2014.06.015

[b10] Zhang Q., Folta K. M. & Davis T. M. Somatic embryogenesis, tetraploidy, and variant leaf morphology in transgenic diploid strawberry (Fragaria vesca subspecies vesca ‘Hawaii 4′). BMC Plant Biol 14, 23 (2014). 24418064 10.1186/1471-2229-14-23 PMC3898059

[b11] Brown T. & Wareing P. Genetical control of everbearing habit and 3 other characters in varieties of Fragaria vesca. Euphytica 14, 97–112 (1965).

[b12] Davis T. M. & Yu H. A linkage map of the diploid strawberry, Fragaria vesca. J. Hered. 88, 215–221 (1997).

[b13] Deng C. & Davis T. M. Molecular identification of the yellow fruit color (c) locus in diploid strawberry: a candidate gene approach. Theor. Appl. Genet. 103, 322 (2001).

[b14] Crozier A. . In Plant Secondary Metabolites: Occurrence, Structure and Role in the Human Diet (2008).

[b15] Urrutia M., Schwab W., Hoffmann T. & Monfort A. Genetic dissection of the (poly)phenol profile of diploid strawberry (Fragaria vesca) fruits using a NIL collection. Plant Sci. Int. J. Exp. Plant Biol. 242, 151–168 (2016). 10.1016/j.plantsci.2015.07.019 26566833

[b16] Almeida J. R. M. . Characterization of major enzymes and genes involved in flavonoid and proanthocyanidin biosynthesis during fruit development in strawberry (Fragaria xananassa). Arch. Biochem. Biophys. 465, 61–71 (2007). 17573033 10.1016/j.abb.2007.04.040

[b17] Albert N. W. . A conserved network of transcriptional activators and repressors regulates anthocyanin pigmentation in eudicots. Plant Cell 26, 962–80 (2014). 24642943 10.1105/tpc.113.122069 PMC4001404

[b18] Ramsay N. A. & Glover B. J. MYB-bHLH-WD40 protein complex and the evolution of cellular diversity. Trends Plant Sci 10, 63–70 (2005). 15708343 10.1016/j.tplants.2004.12.011

[b19] Butelli E. . Retrotransposons control fruit-specific, cold-dependent accumulation of anthocyanins in blood oranges. Plant Cell 24, 1242–55 (2012). 22427337 10.1105/tpc.111.095232 PMC3336134

[b20] Schwinn K. . A small family of MYB-regulatory genes controls floral pigmentation intensity and patterning in the genus Antirrhinum. Plant Cell 18, 831–51 (2006). 16531495 10.1105/tpc.105.039255 PMC1425845

[b21] Butelli E. . Enrichment of tomato fruit with health-promoting anthocyanins by expression of select transcription factors. Nat Biotechnol 26, 1301–8 (2008). 18953354 10.1038/nbt.1506

[b22] Cermak T., Baltes N. J., Cegan R., Zhang Y. & Voytas D. F. High-frequency, precise modification of the tomato genome. Genome Biol 16, 232 (2015). 26541286 10.1186/s13059-015-0796-9 PMC4635538

[b23] Tuan P. A. . The crucial role of PpMYB10.1 in anthocyanin accumulation in peach and relationships between its allelic type and skin color phenotype. BMC Plant Biol 15, 280 (2015). 26582106 10.1186/s12870-015-0664-5 PMC4652394

[b24] Medina-Puche L. . MYB10 plays a major role in the regulation of flavonoid/phenylpropanoid metabolism during ripening of Fragaria × ananassa fruits. J. Exp. Bot. 65, 401–417 (2014). 24277278 10.1093/jxb/ert377

[b25] Lin-Wang K. . An R2R3 MYB transcription factor associated with regulation of the anthocyanin biosynthetic pathway in Rosaceae. BMC Plant Biol 10, 50 (2010). 20302676 10.1186/1471-2229-10-50 PMC2923524

[b26] Lin-Wang K. . Engineering the anthocyanin regulatory complex of strawberry (Fragaria vesca). Front Plant Sci 5, 651 (2014). 25477896 10.3389/fpls.2014.00651 PMC4237049

[b27] Zhang Y. . Transcript quantification by RNA-Seq reveals differentially expressed genes in the red and yellow fruits of Fragaria vesca. PLoS One 10, e0144356 (2015). 26636322 10.1371/journal.pone.0144356 PMC4670188

[b28] Conesa A. . Blast2GO: a universal tool for annotation, visualization and analysis in functional genomics research. Bioinforma. Oxf. Engl. 21, 3674–3676 (2005). 10.1093/bioinformatics/bti610 16081474

[b29] Li S. Transcriptional control of flavonoid biosynthesis. Plant Signal. Behav. 9, e27522 (2014). 24393776 10.4161/psb.27522 PMC4091223

[b30] Duchesne A. N. Histoire naturelle des fraisiers. (Didot le jeune, 1766).

[b31] Iwata H. . The TFL1 homologue KSN is a regulator of continuous flowering in rose and strawberry. Plant J 69, 116–25 (2012). 21895811 10.1111/j.1365-313X.2011.04776.x

[b32] Koskela E. A. . Mutation in TERMINAL FLOWER1 reverses the photoperiodic requirement for flowering in the wild strawberry Fragaria vesca. Plant Physiol 159, 1043–54 (2012). 22566495 10.1104/pp.112.196659 PMC3387692

[b33] Haun W. J. . The Composition and origins of genomic variation among individuals of the soybean reference Cultivar cultivar Williams 82. Plant Physiol. 155, 645–655 (2011). 21115807 10.1104/pp.110.166736 PMC3032456

[b34] Ravi M. & Chan S. W. Haploid plants produced by centromere-mediated genome elimination. Nature 464, 615–8 (2010). 20336146 10.1038/nature08842

[b35] Kanei-Ishii C. . The tryptophan cluster: a hypothetical structure of the DNA-binding domain of the myb protooncogene product. J. Biol. Chem. 265, 19990–19995 (1990). 2246275

[b36] Ogata K. . Solution structure of a DNA-binding unit of Myb: a helix-turn-helix-related motif with conserved tryptophans forming a hydrophobic core. Proc. Natl. Acad. Sci. 89, 6428–6432 (1992). 1631139 10.1073/pnas.89.14.6428 PMC49514

[b37] Schaefer H. M., Levey D. J., Schaefer V. & Avery M. L. The role of chromatic and achromatic signals for fruit detection by birds. Behav. Ecol. 17, 784–789 (2006).

[b38] McKenna A. . The Genome Analysis Toolkit: a MapReduce framework for analyzing next-generation DNA sequencing data. Genome Res. 20, 1297–1303 (2010). 20644199 10.1101/gr.107524.110 PMC2928508

[b39] Cingolani P. . A program for annotating and predicting the effects of single nucleotide polymorphisms, SnpEff. Fly (Austin) 6, 80–92 (2012). 22728672 10.4161/fly.19695 PMC3679285

[b40] Goodstein D. M. . Phytozome: a comparative platform for green plant genomics. Nucleic Acids Res. 40, D1178–D1186 (2012). 22110026 10.1093/nar/gkr944 PMC3245001

[b41] Li H. & Durbin R. Fast and accurate short read alignment with Burrows–Wheeler transform. Bioinformatics 25, 1754–1760 (2009). 19451168 10.1093/bioinformatics/btp324 PMC2705234

[b42] Li H. . The sequence alignment/map format and SAMtools. Bioinforma. Oxf. Engl. 25, 2078–2079 (2009). 10.1093/bioinformatics/btp352 PMC2723002 19505943

[b43] Quinlan A. R. & Hall I. M. BEDTools: a flexible suite of utilities for comparing genomic features. Bioinformatics 26, 841–842 (2010). 20110278 10.1093/bioinformatics/btq033 PMC2832824

[b44] Cheong W.-H., Tan Y.-C., Yap S.-J. & Ng K.-P. ClicO FS: an interactive web-based service of Circos. Bioinforma. Oxf. Engl. 31, 3685–3687 (2015). 10.1093/bioinformatics/btv433 PMC4817113 26227146

[b45] Krzywinski M. . Circos: An information aesthetic for comparative genomics. Genome Res. 19, 1639–1645 (2009). 19541911 10.1101/gr.092759.109 PMC2752132

[b46] Van Bel M. . Dissecting plant genomes with the PLAZA comparative genomics platform. Plant Physiol. 158, 590–600 (2012). 22198273 10.1104/pp.111.189514 PMC3271752

[b47] Schultz J., Milpetz F., Bork P. & Ponting C. P. SMART, a simple modular architecture research tool: Identification of signaling domains. Proc. Natl. Acad. Sci. 95, 5857–5864 (1998). 9600884 10.1073/pnas.95.11.5857 PMC34487

[b48] Tennessen J. A., Govindarajulu R., Ashman T.-L. & Liston A. Evolutionary origins and dynamics of octoploid strawberry subgenomes revealed by dense targeted capture linkage maps. Genome Biol. Evol. 6, 3295–3313 (2014). 25477420 10.1093/gbe/evu261 PMC4986458

[b49] Curtis M. D. & Grossniklaus U. A Gateway cloning vector set for high-throughput functional analysis of genes in planta. Plant Physiol. 133, 462–469 (2003). 14555774 10.1104/pp.103.027979 PMC523872

[b50] Hoffmann T., Kalinowski G. & Schwab W. RNAi-induced silencing of gene expression in strawberry fruit (Fragaria × ananassa) by agroinfiltration: a rapid assay for gene function analysis. Plant J. 48, 818–826 (2006). 17092319 10.1111/j.1365-313X.2006.02913.x

