# Supplementary material for: Genome-scale DNA variant analysis and functional validation of a SNP underlying yellow fruit color in wild strawberry
Source: Sci Rep. 2016 Jul 5;6:29017. doi: 10.1038/srep29017 (PMC4932534; doi:10.1038/srep29017)
Supplement: Supplementary Information [file srep29017-s1.pdf]

Supplementary Information

Title: Genome-scale DNA variant analysis and functional validation of a SNP underlying yellow fruit color in wild strawberry

Charles Hawkins[+], Julie Caruana[+], Erin Schiksnis, and Zhongchi Liu*

Dept. of Cell Biology and Molecular Genetics
University of Maryland
College Park, MD 20742

+: These authors contributed equally to this work

*Corresponding author:
Zhongchi Liu
Phone: (301) 405-1586
Fax: (301) 314-1248
Email: zliu@umd.edu

Supplementary Figures

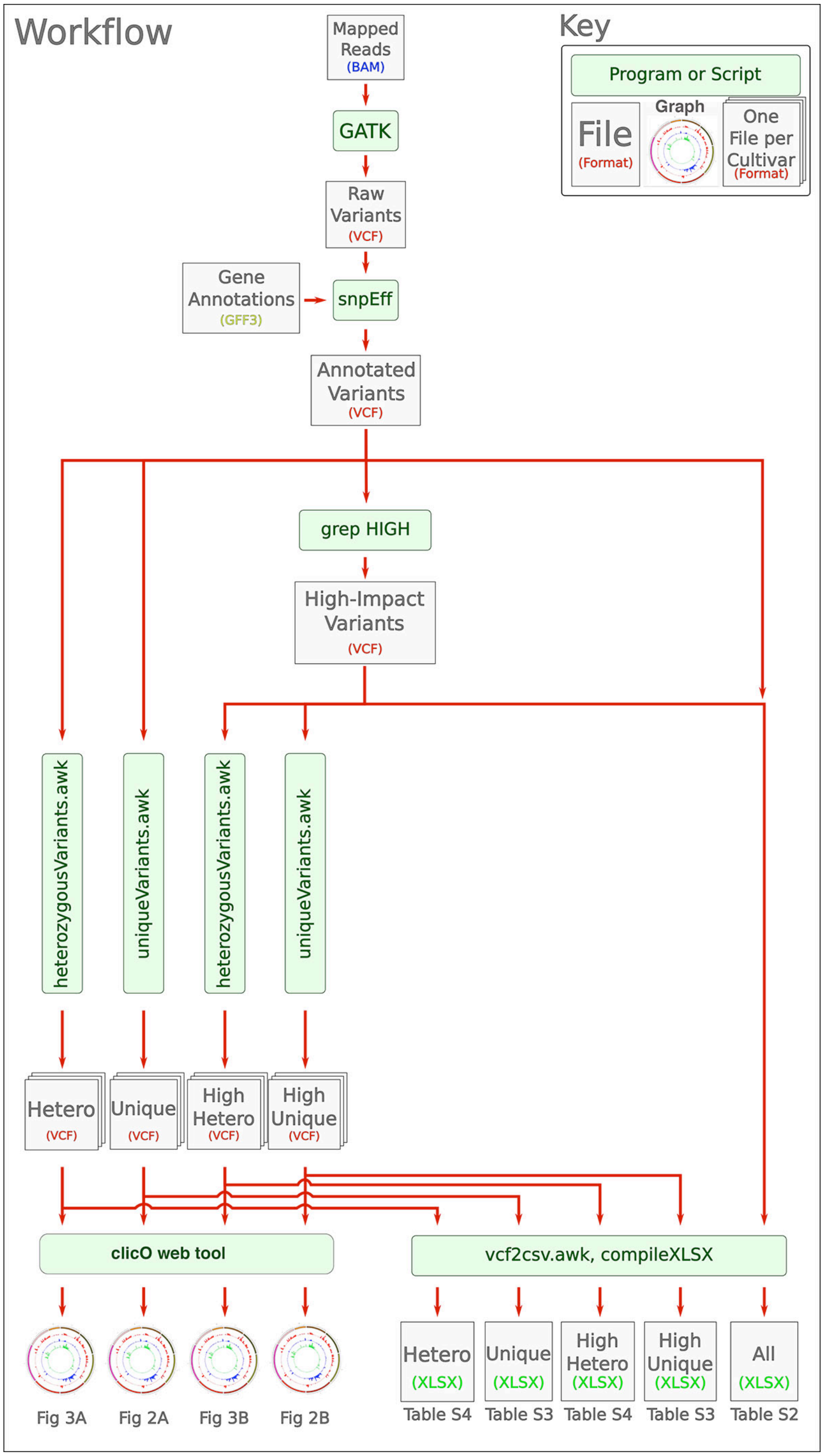

Figure S1. Sequence analysis workflow.

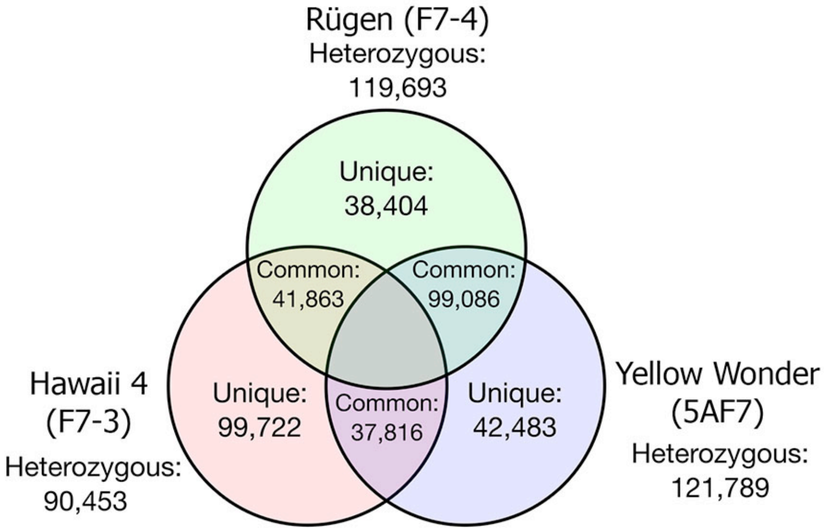

Figure S2. Venn Diagram showing unique and overlapping SNPs found among the three accessions. Number of heterozygous loci in each accession is also indicated.

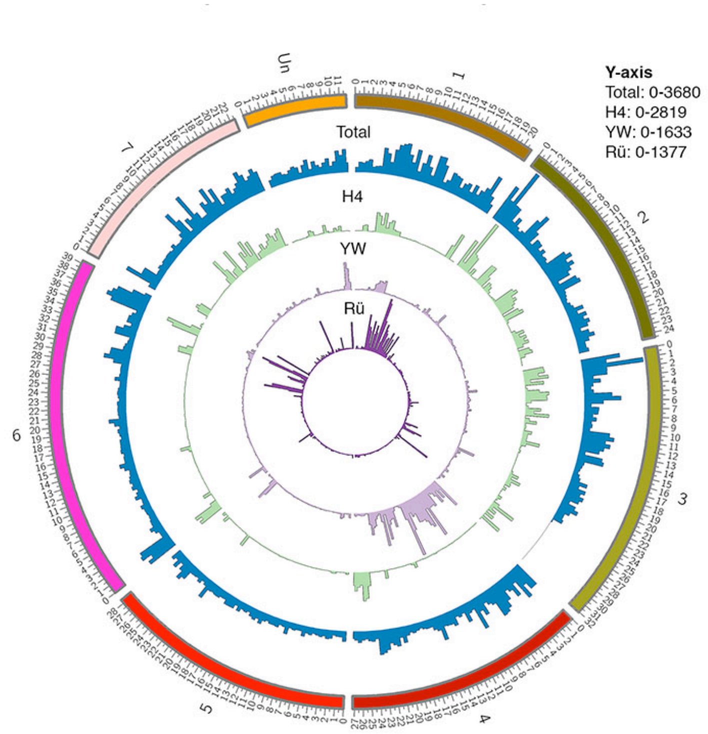

Figure S3. Circos ideogram showing genome-wide distribution of total combined variants in comparison to Accession-Unique variants.

The outermost circle represents the seven *F. vesca* Linkage Groups plus the unanchored scaffolds (Un), drawn to scale in Mbp. The blue track is the histogram showing the distribution of total combined variants (both unique and shared variants) from all three accessions. The inner three tracks are histograms showing distribution of accession-unique variants, which are identical to Figure 2A. Bin size is 500 kb, Y-axis is the number of total variants or variety-unique variants.

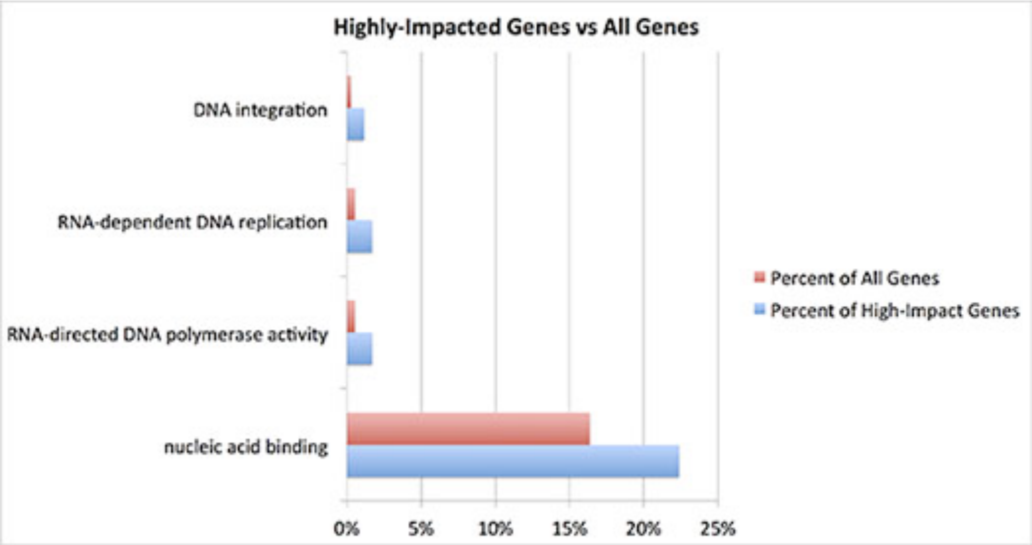

Figure S4. Enriched GO terms among genes (test set) affected by high impact variants in all three accession. The reference set was derived from Phytozome, representing % genes belonging to each GO category.

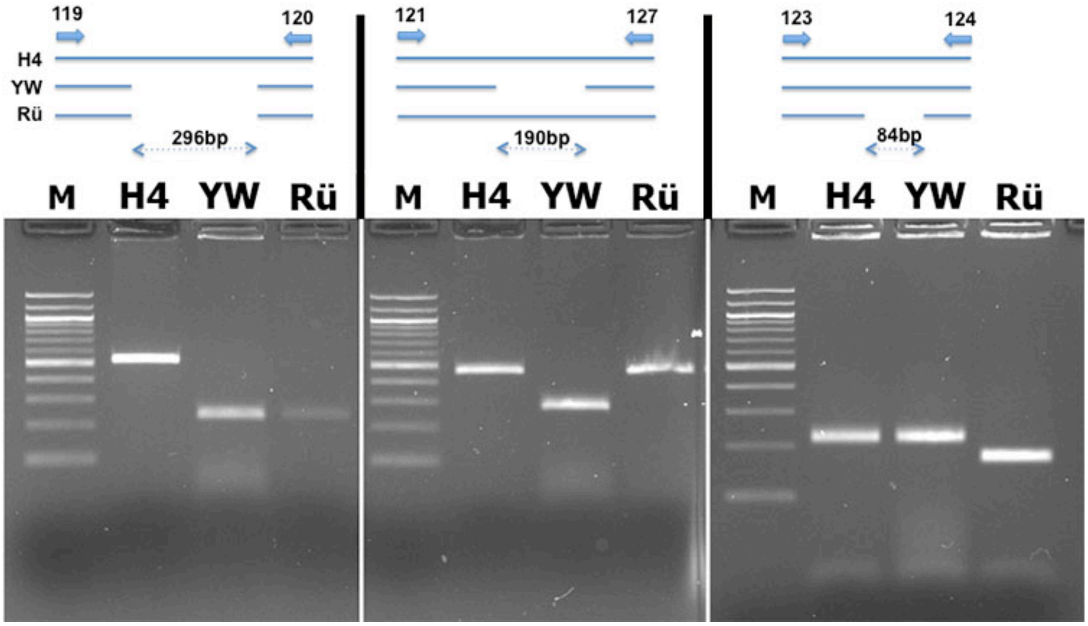

Figure S5. Indel markers that distinguish the three *F. vesca* accessions. Three Indel markers have been developed. Corresponding PCR primer pairs 119/120 (LG2), 121/127 (LG4), and 123/124 (LG6) are respectively indicated as small arrows. Dotted arrow indicates deletion. The agarose gel images show variety-specific banding pattern. Genomic DNA from H4, YW, and Rü served as PCR templates. M is the 100 bp ladder.

Supplementary Tables

Table S1. Read Mapping Metrics.

Variable	H4 (F7-3)	Rü (F7-4)	YW (5AF7)	Average	Total	Percent
**Total Reads**	96,788,229	71,040,883	75,226,315	81,018,476	243,055,427	
**Unmapped Reads**	9,675,731	8,959,117	9,067,735	9,234,194	27,702,583	
**Percent Unmapped**	10.0%	12.6%	12.1%	11.6%		11.4%
**Duplicate Reads**	16,886,729	11,454,393	12,081,324	13,474,149	40,422,446	
**Percent Duplicate**	17.4%	16.1%	16.1%	16.5%		16.6%

Table S2. Candidate Gene Summary.

Family	Gene ID	Gene Name	Variant	Analysis
**3GT**	gene28059	Anthocyanidin 3-O-glucosyltransferase 5 (probable)	N416S	Not Significant (Polar to Polar)
			L271F	Not Significant (Nonpolar to Nonpolar)
	gene30501	Anthocyanidin 5,3-O-glucosyltransferase (putative)	V356A	Not Significant (Nonpolar to Nonpolar)
**Myb**	**gene01311**	Transcription factor MYB32 (AtMYB32) (similar to)	N348K	**Significant (Basic to Polar)**
	gene13016	Myb-like protein H (probable)	H174R	Not Significant (Basic to Basic)
	gene24027	Transcription factor MYB44 (AtMYB44) (probable)	T306S	Not Significant (Polar to Polar)
	**gene24516**	Transcription factor MYB21 (AtMYB21) (similar to)	G65V	**Significant (Nonpolar to Polar)[1]**
	gene28021	Myb-related protein 315 (probable)	E178D	Not Significant (Acidic to Acidic)
	**gene31413**	Transcription factor MYB113 (AtMYB113) (similar to)	W12S	**Significant (Polar to Nonpolar)**

Table S3. Indel Markers That Distinguish Each Variety

Marker unique to	Forward primer sequence 5' – 3'	Reverse primer sequence 5' – 3'	Location in Genome	Deletion length (accession)	Amplicon length in H4	Amplicon length in YW	Amplicon length in Rü
H4 (119/120)	CTCTTAGACACATCATCTGCATACGG	GATGAAGGATGACTGAACTATTACATAGG	LG2 12558999 - 12559294	296 (YW, R)	535	239	239
YW (121/127)	CATGAGTTGTAGGTGAGCCGTGGCT	TGGATAAGGAGAATGCCTCAATGTGGTGC	LG4 5350967 – 5351156	190 (YW)	467	377	467
Rü (123/124)	GGGATCGAGTTCAGCAATGCTTGA	ACTTTCCCATTTTGGAGCCTGGG	LG6 28793423 – 28793506	80 (R)	260	260	180

Supplementary Methods

The following is a listing of the various scripts written to perform the data analysis presented in this paper. These scripts were run on Mac OS X for this paper and all should work on most Linux systems without modification provided the necessary tools are installed (Awk, Perl, Zsh, and R). Running them on Windows systems may require modification or the installation of a Unix environment.

VCF Filtering Scripts

The scripts in this category will take .vcf files and extract out only the lines matching the script's criteria. Most of them operate under the assumption that there are exactly three cultivars being analyzed and may not work for a different number of samples. They all use awk. Arguments are generally given to awk scripts using -v variable=value. These are all able to work with .eff.vcf files from snpEff. Most of these scripts take one or two sample numbers (from 0 to 2) to indicate which cultivar(s) you want them to act on. In our .vcf files the cultivars are numbered as follows:
0 = Hawaii 4
1 = Rügen
2 = Yellow Wonder

uniqueVariants.awk

This script extracts .vcf lines denoting loci where the given cultivar has no allele that is also found in either of the other two varieties. The variety to analyze may be specified by setting the "sample" variable.

```
./uniqueVariants.awk -v sample=0 infile.vcf >
outfile.vcf
```

The above will take the lines from infile.vcf where there is an allele unique to Hawaii 4 (sample 0) and save them in outfile.vcf.

heterozygousVariants.awk

This script extracts .vcf lines denoting loci where the given variety is heterozygous (has two or more alleles). The variety to analyze may be specified by setting the "first" variable.

```
./heterozygousVariants.awk -v first=2 infile.vcf
> outfile.vcf
```

The above will take the lines from infile.vcf where Yellow Wonder (sample 2) is heterozygous and save them in outfile.vcf.

homozygousVariants.awk

This script extracts .vcf lines denoting loci where the given variety is homozygous (has only one allele). The variety to analyze may be specified by setting the "first" variable.

```
./homozygousVariants.awk -v first=0 infile.vcf >
outfile.vcf
```

The above will take the lines from infile.vcf where Hawaii 4 (sample 0) is homozygous and save them in outfile.vcf.

differingVariants.awk
This script extracts .vcf lines denoting loci where the two given varieties differ (do not share any alleles). The varieties to analyze may be specified by setting the "first" and “second” variables.

```
./differingVariants.awk -v first=2 -v second=1
infile.vcf > outfile.vcf
```

The above will take the lines from infile.vcf where Yellow Wonder (sample 2) and Rügen (sample 1) do not share any alleles and save them in outfile.vcf.

commonVariants.awk
This script extracts .vcf lines denoting loci where the two given cultivars are the same, meaning that they are both homozogous for the same allele and do not share this allele with the remaining cultivar. The varieties to analyze may be specified by setting the "first" and “second” variables.

```
./commonVariants.awk -v first=0 -v second=1
infile.vcf > outfile.vcf
```

The above will take the lines from infile.vcf where Hawaii 4 (sample 2) and Rügen (sample 1) are homozygous for the same allele and this allele is not found in Yellow Wonder. It will save these lines in outfile.vcf.

VCF to CSV
The following scripts both extract information from a .vcf file and save it to a .csv file.

vcf2csv.awk
This extracts the chromosome, location, and variant list for each cultivar for each variant and saves them to a .csv file that can be opened in a spreadsheet program. Each cultivar has a column in the .csv file that lists the sequences of the alleles it has, separated with a vertical bar ("|") if more than one is present.

```
./vcf2csv infile.vcf > outfile.csv
```

This script was used in the generation of the supplemental Excel files in the paper.

extractLocs

This script is similar to the above but will only extract the chromosome and location of each .vcf line. Its output is intended for use in generating the histograms using the R scripts below. It requires Zsh to be installed (which it is by default in Mac OS but may not be in some Linux systems).

```
./extractLocs infile.vcf > outfile.csv
```

Fixing a GFF file for use with snpEff

fix-gff.awk
This script will fix a Phytozome .gff3 file so that snpEff can read it. The Fvesca_226_genes.gff3 file taken from Phytozome had the frame offset field for each exon calculated in a way different from what snpEff expects, and so snpEff did not interpret it correctly. This script was written to process the .gff3 file so as to correct this incompatibility. It is not guaranteed to work with other .gff files. The input and output should not be the same file.

```
./fix-gff.awk infile.gff > outfile.gff
```

Removing variants in high-read regions

The scripts in this category are used to remove variants that are in regions where the reads are too high.

extractHCRanges.awk

This script is the first stage of the process of cutting out variants that are in regions where the read count is too high (any such region likely represents a sequencing anomaly and all variants found there should therefore be considered unreliable). The task of this first script is to mark the regions of the genome within which variants should be excluded. It takes as input a coverage file produced by the genomeCoverageBed utility found in BedTools (http://bedtools.readthedocs.org). This utility should be run on the final .bam file, the one that is also fed into the variant calling software. Run it with the -bg and -ibam options, as in this example for Hawaii 4:

```
genomeCoverageBed -bg -ibam Hawaii4Recal.bam >
Hawaii4.bg
```

This will produce the file Hawaii4.bg which can be fed into extractHCRanges.awk. A read cutoff may be specified to extractHCRanges.awk with -v cutoff=50 (50 was the cutoff used for the analysis described in this paper). To continue the same Hawaii 4 example:

```
extractHCRanges.awk -v cutoff=50 Hawaii4.bg >
Hawaii4.HC50.csv
```

This will produce a .csv file to be fed into cutReads (described below) to specify which regions the latter script should cut. Each row in this file represents a region wherein the coverage exceeds the cutoff, and has three fields: the chromosome, the start of the region in bp, and the end of the region in bp. The latter two are inclusive (i.e. the region LG1,1,10 would include bases 1 and 10 on the chromosome LG1)

cutReads

This script will take a file that lists variants and will remove those entries that fall within any region marked in the csv file produced by extractHCRanges.awk. It can cut both VCF files and variant-containing CSV files of the kinds produced by vcf2csv.awk and extractLocs (described above). It requires the Perl libraries Getopt::Long and List::Util 1.33, both available on CPAN. The command-line options are as follows:

- --zones | -z: Specify the zones file, the CSV file produced by extractHCRanges.awk
- --varsfile | -v: Specify the file containing the variants to be cut using the zones file
- --filetype | -f: Specify as “csv” if the variants file is a CSV file or “vcf” if it is a VCF file. Default is “csv”

Continuing our Hawaii 4 example, we can cut a VCF file with:

```
cutReads -v H4-Het.vcf -z Hawaii4.HC50.csv -f vcf
> Hawaii4.Het.HC50.vcf
```

Or we could cut a CSV with:

```
cutReads -v H4-Het.csv -z Hawaii4.HC50.csv >
Hawaii4.Het.HC50.csv
```

Converting CSV to XLSX

compileXlsx

This Perl script converts and compiles a set of .csv files into a set of multi-sheet Excel (.xlsx) documents. It requires the Perl libraries Excel::Writer::XLSX, Text::CSV, and Switch. A configuration file, itself in CSV format, is used to specify the names of the Excel workbooks, the name of each sheet in each workbook, and the name of the .csv file whose contents should be placed in each sheet. The configuration file should consist of single-column rows with excel file names, each followed by one or more two-column rows wherein the first column is the name to be given to a sheet and the second column is the name of the .csv file whose contents should be placed in that sheet. The first row of each .csv file is presumed to be column labels and is therefore styled bold in the Excel file. The name of the configuration file should be the sole

argument given to compileXlsx.

```
compileXlsx config.csv
```
